# Obstacle Detection as a Safety Alert in Augmented Reality Models by the Use of Deep Learning Techniques

**DOI:** 10.3390/s17122803

**Published:** 2017-12-04

**Authors:** Dawid Połap, Karolina Kęsik, Kamil Książek, Marcin Woźniak

**Affiliations:** Institute of Mathematics, Silesian University of Technology, Kaszubska 23, 44-100 Gliwice, Poland; Dawid.Polap@polsl.pl (D.P.); Karola.Ksk@gmail.com (K.K.); kamilksiazek95@gmail.com (K.K.)

**Keywords:** convolutional neural network, spiking neural network, hybrid architecture, obstacle detection, augmented reality

## Abstract

Augmented reality (AR) is becoming increasingly popular due to its numerous applications. This is especially evident in games, medicine, education, and other areas that support our everyday activities. Moreover, this kind of computer system not only improves our vision and our perception of the world that surrounds us, but also adds additional elements, modifies existing ones, and gives additional guidance. In this article, we focus on interpreting a reality-based real-time environment evaluation for informing the user about impending obstacles. The proposed solution is based on a hybrid architecture that is capable of estimating as much incoming information as possible. The proposed solution has been tested and discussed with respect to the advantages and disadvantages of different possibilities using this type of vision.

## 1. Introduction

Augmented reality (AR) is no more than an expansion of reality with virtual elements that interact with some senses that comprise the perceptions of the user. The idea of integrating the real world with some virtual extensions has been a breakthrough in current technology. The simplicity of acquisition and the huge possibilities for facilitating various applications in our lives are the main advances. Recently, the solutions have been primarily used in mobile platforms as new aspects in various games. Sensors and devices transfer actions from the user into the game engine that reacts to these over virtual extensions. These man-machine interactions extend human senses and let users experience various stimuli from the virtual world that make the game more real and interesting. This type of interaction contributes to modifications in our lives. In particular, such games encourage children to leave the house and play in the open air while staying in contact with their favorite electronics. This represents a big change to modern lifestyle and reality, where many consoles and computers simply keep children at home in front of monitor. One of the most popular games based on AR is *Pokemon GO*, which has contributed to the popularization of this technology among young people. The authors of [1] presented how AR can improve state of health for children with social withdrawal, and the authors of [2] discussed how participation in *Pokemon GO* can encourage children to undertake more physical activity outside. Aspects of the popularity of this game were discussed in [3], where the authors discussed different aspects, including social, emotional, hedonic, and even nostalgic factors. As the main limitation, physical risk is pointed out.

Another aspect of AR is its excellent contribution to educational applications. In [4] the authors presented the use of AR as a tool in learning processes. The proposed idea discussed benefits from the creation of virtual models of different parts of human body in order to use them for explanation of the structure and working of tissues. Another idea with respect to the didactic application of AR was shown in [5]. The authors undertook this project in a primary school with teachers and students. They created a game which helps to discover the significance of historical buildings in Greece. Also, in [6], educational aspects of AR were discussed for more efficient learning processes. The merit was in creating a game that was useful in astronomy classes. However, AR is not only useful for educational purposes, but also medical ones. This technology can be used for assistance during surgery. One such example is presented in [7], where the automatic localization of the endoscope in intraoperative computed tomography. images was presented. AR for reducing the problem of childhood obesity was described in [8]. An interesting application is to use AR for tourists as a general guide [9]. Moreover, AR can be used to assist disabled people. One such proposition was described in [10,11] as a system for the visually impaired with the use of this technology. Another example is found in [12,13] where robotic aspects were analyzed. This example was very useful for wheelchairs in a circular environment.

### Related Works

Despite so many varied possibilities for applications, currently, gaming is the most developed area. Here, AR can exceed the imposed limits by enhancing playability as well as the perceptions of players. In [14] the game in AR was described as application dedicated to smartphones. The action may take place in streets or other public places that can move users far from digital screens. Other applications in mobile devices were presented in [15]. This article demonstrates the AR–Zombie game, where a player has to kill virtual zombies displayed on the screen. Players distinguish between visible creatures by using a face recognition system which intensifies player interactions and experiences. Some other augmented reality touch-less games are shown in [16]. A multiplayer game called Robot Devastation was described in [17]. To play it is necessary to have an inexpensive robot and a PC. AR was also applied in a mobile game called Rediscovering Daereungwon [18]. This idea assists tourists exploring historic Korean places by integration of the Memorable Experience Design and Interest Curve. In [19] authors extended geocaching possibilities in augmented reality. This allows users to learn new information about historic landmarks en route and take part in games providing guidance on the next steps of the search. An interesting concept was presented in [20], where it was proposed that traditional trading cards be replaced with Stereo Cards based on augmented reality. These new cards are identified by electronic devices (for instance digital cameras) where makers describe their usage. Of course, games that are played in real-time in the real world involve many dangers caused by the inattention of the players. A player who is lost in a game and wants to cross the road can forget to look if a car is coming. Another risk may be when a player has to move and react quickly to a variety of actions. This can cause some accidents, like falling over a low fence, tree, or lantern. All of these situations are related to certain obstacles that occur when playing in the real world. Therefore, intelligent sensing technologies are useful in various detection systems constructed for ad hoc networks [21] and vehicle parking slots [22].

In this article, the idea of obstacle detection based on deep learning methods is presented. We present a system based on AR in which implemented methods work as detectors of obstacles on the way. The proposed architecture uses not only video but also real-time information processing from mobile devices that support AR technology. The presented solution has been tested on a dedicated application and the results show high efficiency in detection over various objects placed in different landscapes. The technology is easy to transfer to more sophisticated applications that can support elder people or work in complex systems for unmanned vehicles. The novelty of this solution is the possibility of interacting in real-time with humans or other devices by suggesting other routes and warning about possible accidents. The system can work in motion at various speeds. The composition of detection mechanics is based on a complex structure of convolutional and spiking neural networks which are used as detectors of various aspects of moving objects. The information over the path is evaluated in different parts of the system, where each of the structures focuses on other aspects of the input. This complex structure is novel, and due to the particular detection advances of these structures is efficient over various obstacles.

## 2. Capturing Data in Environment

Perception in augmented reality is accompanied by some electronic devices—in most cases mobile phones with installed software allowing a view of the modified environment. The simplest model of the AR application requires a camera, a microphone, and additional sensors that are built into today’s mobile phones. To obtain the most spectacular effect, a lot of information from the environment is needed for the analysis of the surrounding world. In this section, the data and the feature extraction processes from each of sensors are presented.

### 2.1. Data Extraction from the Camera Registry

The main advantage of the AR technology is obtained by the sensor of the camera, which transmits the image to the display. When the user moves, objects in the real environment move with him/her or move away. The display expands or reduces these objects in the transmitted image. Captured video is mostly composed of 24 frames per second. The analysis of each frame would require a huge number of calculations. This would make it impossible to use in real time. To avoid this, in the proposed solution one frame is taken every 2 s. User movement in a given time interval can indicate the location of the objects. Frame analysis is based on finding key points, reducing their number, and ultimately determining whether any of visible objects is the obstacle. Each frame is analyzed by the SURF (*Speeded up robust features*) algorithm [23]. The algorithm is based on calculating the Hessian matrix to locate the important pixels with the neighborhood. This matrix is represented as:

(1) $$H\left(x,\omega \right)=\left[\begin{array}{cc} L_{xx}\left(x,\omega \right) & L_{xy}\left(x,\omega \right)\\ L_{xy}\left(x,\omega \right) & L_{yy}\left(x,\omega \right) \end{array}\right]$$

where $L_{ii}\left(i,\omega \right)$ is an image convolution of I(x) with the Gaussian second derivative (using Gaussian kernel g(ω)) which can be formulated as:

(2) $$L_{xx}\left(x,\omega \right)=I\left(x\right)\frac{\partial^{2}}{\partial x^{2}}g\left(\omega \right)$$

(3) $$L_{yy}\left(x,\omega \right)=I\left(x\right)\frac{\partial^{2}}{\partial y^{2}}g\left(\omega \right)$$

(4) $$L_{xy}\left(x,\omega \right)=I\left(x\right)\frac{\partial^{2}}{\partial xy}g\left(\omega \right)$$

In the above formulas, the argument *x* is the sum of all pixels in the neighborhood of the analyzed point defined as:

(5) $$I\left(x\right)=\sum_{i=0}^{i\leq x}\sum_{j=0}^{j\leq y}I\left(x,y\right)$$

With all these formulas, the SURF algorithm detects the important points using non-maximal suppression of the matrix determinant calculated using the following equation:

(6) $$det\left(H_{approximate}\right)=D_{xx}D_{xy}-{\left(wD_{xy}\right)}^{2}$$

where $D_{xx}$ indicates $L_{xx}\left(x,\omega \right)$, and *w* is the weight. All the points that can be described as the extremes are marked as key points.

Of course, the number of found points is usually very large. To minimize the number, we delete all points that do not have neighbors in the radius *r*. The radius of the neighborhood should depend on the size of the image (supposing the width is marked as *w* and height as *h*). Moreover, when the radius is too large, it may leave points that are not significant in the vicinity of the various objects. Using all these facts, the radius can be calculated as:

(7) $$r=\left\lceil \sqrt{\frac{w+h}{\pi }}\right\rceil$$

The minimized number of points can be grouped into certain clusters of objects. We understand as clusters the set of points $\left\{x_{0},\ldots ,x_{n}\right\}$ where each element has at least one neighbor in that set. Each cluster will be evaluated with respect to the changing intensity between two frames. We understand the intensity as follows:

(8) $$\begin{array}{c} \Phi \left(\{x_{0},\ldots ,x_{n}\}\right)=\Phi \left(x\right)=\frac{\max\left(x^{width}\right)-\min\left(x^{width}\right)}{2}\\ +\frac{\max\left(x^{height}\right)-\min\left(x^{height}\right)}{2} \end{array}$$

Hence, the defined intensity can be used as a tool to check what has changed in the environment. An increasing or decreasing value of intensity in particular areas is caused by the movement of the object or the user. In our consideration, we want to search for obstacles, so we will be interested in objects that are approaching. Therefore, for our purposes the intensity comparison process for searching obstacles can be illustrated as:

(9) $$\left\{\begin{array}{c} \begin{array}{c} \Phi_{i}\left({\left\{x_{0},\ldots ,x_{n}\right\}}_{n-1}\right)\leq \Phi_{i}\left({\left\{x_{0},\ldots ,x_{n}\right\}}_{n}\right)\\ \mathrm{approaching}\;\mathrm{obstacle} \end{array}\\ \begin{array}{c} \Phi_{i}\left({\left\{x_{0},\ldots ,x_{n}\right\}}_{n-1}\right)>\Phi_{i}\left({\left\{x_{0},\ldots ,x_{n}\right\}}_{n}\right)\\ \mathrm{receding}\;\mathrm{obstacle} \end{array} \end{array}\right.$$

where *i* means a cluster number, and *n*, and n−1 mean the *n*-th and n−1-th frames, respectively.

### 2.2. Data Extraction from the Sound Registry

Analysis of sounds from the environment may prevent various problems with oncoming cars or other objects. In the ideal situation, the driver uses a horn on a pedestrian who does not pay attention to what is going on around him/her. Quite often, drivers listen to loud music, which can also inform about the approaching vehicle. It is the same with privileged cars that use continuous sound when on duty throughout the day. Quick detection may allow for reporting any nearby danger to users.

The audio signal *s* is a wave that can be defined as follows:

(10) $$s\left(t\right)=\sum_{i=1}^{N}A_{i}\left(t\right)\sin\left[2\pi F_{i}\left(t\right)t+\omega_{i}\left(t\right)\right]$$

where *t* is time, A(t) is the amplitude, F(t) means frequency, and ω(t) is a phase. Unfortunately, this form cannot be analyzed. Hence, some transform is needed. The most popular and well known is the short-time Fourier transform (STFT) [24] described in discrete form as:

(11) $$S\left\{s\left[n\right]\right\}\left(m,f\right)=\sum_{n=-\infty }^{\infty }s\left[n\right]w\left[n-m\right]\exp\left(-jfn\right)$$

Application of the STFT gives a discrete signal that can be analyzed and used in different applications. We propose calculating an estimation of energy density in time-space for the possibility of presenting the signal in a 2D graphical representation, more accurately a flattened 3D graph. It is called a spectrogram and can be done by calculating the following formula:

(12) $$spectrogram\left\{s\left(t\right)\right\}\left(t,f\right)\equiv {\left|S\left(t,f\right)\right|}^{2}$$

The flattening of the graph means that for each point (x,y) (where *x* is a representation of time and *y* of frequency), the value is understood as the intensity of that point on the given axes. The intensity is depicted by the shade of color—the darker color, the higher the intensity.

In order to remove as much noise as possible, the graph will be treated as a graphic image that needs to be simplified. Simplification involves the use of simple graphic filters. Assume that the pixel is marked as ϕ and interpreted in an RGB color model. Each of the color components for a given pixel can be described as a function for R(ϕ), G(ϕ), and B(ϕ). To simplify the formulas, we denote ω(ϕ) as each of these components, which can be formulated as ω=R(ϕ)∨ω(ϕ)=G(ϕ)∨ω(ϕ)=B(ϕ). At first, the image is converted to grayscale which consists of replacing each pixel component with the shade of gray defined as:

(13) $$\omega \left(\phi \right)=\frac{R(\phi )+G(\phi )+B(\phi )}{3}$$

The next step is to decrease the gamma value by the so-called gamma correction described as:

(14) $$\omega \left(\phi \right)=255\times {\left(\frac{\omega (\phi )}{255}\right)}^{\gamma },\;\mathrm{where}\;\gamma >0$$

After this step the contrast is increased. For this operation, the contrast correction factor is calculated as:

(15) $$v=\frac{259(\alpha +259)}{255(259-\alpha )}$$

where α is a level of contrast and it is used to calculate the adjustment in contrast by the following formula:

(16) ω(ϕ)=v(ω(ϕ)−128)+128

These filters allow to us simplify the graphics. We have investigated different values of γ and α to find the optimal ones that can be used in general purposes. This is important for the efficiency of AR applications since there are different types of microphones in mobile phones and in addition, the intensity of the recorded sound may vary. The main idea is to simplify the spectrograms in such a way that they can be used as sources of knowledge that describe the environment for later classification. These two values cannot be analyzed separately because there may be a situation when the best results from first filter will be deteriorated by using a second filter, which gives excellent results for the image without the operation of the first one.

To find the best match between the two parameters, different variants have been tested in terms of information quality (pixels) through the entropy equation, where the probability of each pixel remains the same, which can be defined as:

(17) $$H\left(X\right)=\log_{2}\left(n\right)$$

where *X* is a set of pixels and *n* is the number of elements in this set. Of course, this calculation cannot be done for only one sample image. For this purpose, we used 100 grayscale spectrograms and then calculated entropy as:

(18) $$H\left(X\right)=\log_{2}\left(\frac{1}{100}\sum_{i=0}^{100}n_{i}\right)$$

**Figure 1:**
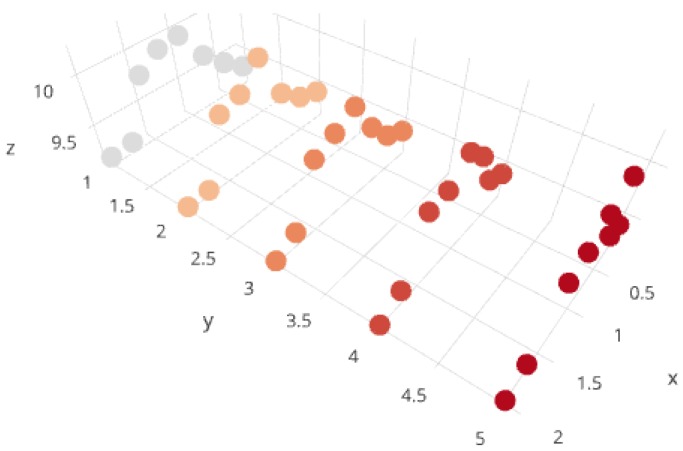
Graph of entropy values for spectrograms with the respect to parameters γ (on OX), α (on OY), and average entropy (on OZ).

Calculated information about entropy is presented in Figure 1 and it is easy to see that the best results are obtained for γ=0.75 and α=5, where the entropy is almost 10.23. Slightly poorer values were obtained for γ=1 and α∈{3,4,5}.

So, these simplified graphics are used as the input features describing the sound in the real-time environment.

### 2.3. Data Extraction from Other Sensors

Mobile phones or tablet have built-in sensors that allow to better analysis of the environment. A few of them can be used in the terms for finding some obstacles in the range of the user. The most important is the gyroscope which is used to measure the position of the device relative to the axes OX, OY, and OX. This provides the opportunity to control various functions and movements in applications by moving the device. In practice, this sensor gives us three values representing the exact position of the device, which can be described as a set of coordinates $\left\{x_{dev},y_{dev},z_{dev}\right\}$. Other built-in sensors are thermometers, hygrometers, and altimeters, which offer data about the temperature, humidity, and altitude above the sea level, respectively. These data can be used in analyzing environmental changes when the user moves. Moreover, in our consideration, rain or fog may limit movement and cause problems during the use of various applications. These weather phenomena reduce the visibility of the player, so the importance of alerting the user to emerging hurdles or problems must be based on other sensors. Such information is supplied by the moisture sensor.

## 3. Neural Techniques

Hybrid solutions are composed as complex structures that have co-working modules made up of specific tasks in the project. The system we present in this article is a complex structure where we have used various models of neural networks, each of which has special properties that makes it more efficient in this implementation.

### 3.1. Spiking Neural Network

A model of the spiking neural network (SNN) architecture simulates voltage change that occurs in the axons of the neurons while transmitting the signal over the tissues in our bodies [25,26]. We name this signal an impulse of the information. From the technical point of view, this impulse is a spike of the voltage that comes from the change in the potential of the neural membrane while sending information to other neurons. Each impulse is passing through whole network and is perceived by specific areas in brain. This kind of stimulation can cause, among other things, memories or processing of received information. A mathematical model of this situation assumes that the impulse is generated after exceeding a threshold value. This type of neural architecture is perfect for processing various signals that vary over time intervald.

The basic unit of the SNN architecture is a neuron. All layers in the SNN are composed of connected neurons that communicate to forward the impulses of information. A set of neurons $\Xi_{j}$ forming the layer *i*-th is connected with another set of neurons from the previous layer *i*-th. The impulse of information is sent over the interlayer connections in time $t_{i}$ if the threshold value ν exceeds the limit level. The state of each neuron $x_{j}\left(t\right)$ defined for the exact time *t* is defined as:

(19) $$x_{j}\left(t\right)=\sum_{i\in \Xi_{j}}w_{ij}\epsilon \left(t-t_{i}\right)$$

where $w_{ij}$ is the connection weight for the neurons *i*-th and *j*-th, and ϵ(t) is an impulse function defined as:

(20) $$\epsilon \left(t\right)=\frac{t}{\tau }\exp\left(1-\frac{t}{\tau }\right)$$

where τ is the constant value that represents membrane potential.

Each of the neurons changes between the reproduction of the impulse and its retrieval. This delay in time is marked as $d^{k}$ for the *k*-th connection and the time between the states is calculated as:

(21) $$y_{j}^{k}\left(t\right)=\epsilon \left(t-t_{j}-d^{k}\right)$$

where the impulse after first exceeding threshold limit ν by neuron $x_{j}\left(t\right)$ was generated in time $t_{j}$.

We calculate the state of neuron $x_{j}$ using Equations (19)–(21) as:

(22) $$x_{j}\left(t\right)=\sum_{i\in \Xi_{j}}\sum_{k=1}^{m}w_{ij}^{k}y_{i}^{k}\left(t\right)$$

The topology of the SNN is similar to classical neural networks: we have the input, multiple hidden layers, and the output.

#### Training

The network must be tuned to process the input, and for this we use algorithms that correct the weights of the connections between the network layers. *SpikeProp* is a devoted method for training the SNN. This method, proposed in [27], is derived from the classic back-propagation algorithm. To describe the procedure we use symbols: *H* for the input, *I* for hidden layers, and *J* for the output.

Input data that enters into the input layer can be described as $\left\{\left[t_{1},\ldots ,t_{h}\right],\ldots \right\}$, and the time to generate the impulse in neuron j∈J as $\left\{t_{j}^{d}\right\}$. The least squares method may be used as error function defined as:

(23) $$E=\frac{1}{2}\sum_{j\in J}{\left(t_{j}^{a}-t_{j}^{d}\right)}^{2}$$

where $t_{j}^{a}$ is the last time the impulse was generated and $t_{j}^{d}$ is called the expected time.

For the training we first calculate the change of the connection weights for the output layer:

(24) $$\Delta w_{ij}^{k}=-\eta y_{i}^{k}\left(t_{j}^{a}\right)\delta_{j}$$

where η is the change coefficient given as a constant value, and $\delta_{j}$ is calculated as:

(25) $$\delta_{j}=\frac{\partial E}{\partial t_{j}^{a}}\frac{\partial t_{j}^{a}}{\partial x_{j}\left(t_{j}^{a}\right)}$$

The change of the connection weights for the hidden layers is:

(26) $$\Delta w_{hi}^{k}=-\eta y_{h}^{k}\left(t_{i}^{a}\right)\delta_{i}$$

where

(27) $$\delta_{i}=\frac{\sum_{j\in \Xi^{i}}\delta_{j}\left[\sum_{k}w_{ij}^{k}\left(\frac{\partial y_{i}^{k}\left(t_{j}^{a}\right)}{\partial t_{i}^{a}}\right)\right]}{\sum_{h\in \Xi^{i}}\sum_{l}w_{hi}^{l}\left(\frac{\partial y_{h}^{l}\left(t_{i}^{a}\right)}{\partial t_{i}^{a}}\right)}$$

### 3.2. Convolutional Neural Network

The convolutional neural network (CNN) proposed in [28] simulates the primary brain cortex. The CNN is devoted to image processing, since the construction of the layers adjusts to the information presented in the images during convolution, pooling, and full processing of the neural units.

The convolution layer extracts information from input images. It is a three-dimensional system of neurons that is composed as an extraction system. Over the width, height, and depth we compute average values of the filters for the input image. The ω filter is run over the image as a matrix of coefficients that blur the content. We use the matrix to change pixel values using the step of *S* pixels. The size of the convolutional layer depends on the size of the image, i.e., for the image size N×N pixels we use filter matrix m×m to calculate the output:

(28) $$s_{output}=\frac{N-m}{S}+1$$

where $s_{output}$ is the output size. As a result we have the feature maps. Next the input neuron $x_{ij}$ on each layer *l*, computes the sum from the previous layer l−1 multiplied by the weight of the filter matrix as:

(29) $$x_{ij}^{l}=\sum_{a=0}^{m-1}\sum_{b=0}^{m-1}\omega_{ab}y_{\left(i+a)(j+b\right)}^{l-1}$$

The pooling (also known as the sub-sampling) layer reduces the size of received images. For this operation we use the maximum value from the pixels in the ω filter of the size a×a. The maximum for each of the filters simply replaces the window, and then we move the filter by a step and again replace all the pixels with the maximum value. This helps to reduce the information from the convolution layer.

Fully connected layers process the information from pooling. This structure is similar to the regular construction of neural networks. Each pixel from pooling is forwarded as a single input to the network. Therefore, the number of neurons is equal to the number of pixels in the image from pooling. The number of the layers consists of assumed fully-connected layers plus the output layer.

#### Training

The CNN structure must be tuned for proper classification. We use the back-propagation algorithm. The error function f(·) is used for correction of the output $\frac{\partial f}{\partial y_{ij}^{l}}$ of the neuron at position i,j in the layer *l*.

We calculate the error $\frac{\partial f}{\partial y_{ij}^{l}}$ for the output layer. Then we use a chain rule to modify connection weights using information gradient for all neurons $x_{ij}^{l}$ as:

(30) $$\frac{\partial f}{\partial \omega_{ab}}=\sum_{i=0}^{N-m}\sum_{j=0}^{N-m}\frac{\partial f}{\partial x_{ij}^{l}}\frac{\partial x_{ij}^{l}}{\partial \omega_{ab}}=\sum_{i=0}^{N-m}\sum_{j=0}^{N-m}\frac{\partial f}{\partial x_{ij}^{l}}y_{\left(i+1)(j+b\right)}^{l-1}$$

The applied information gradient value is calculated using the error value $\frac{\partial f}{\partial x_{ij}^{l}}$:

(31) $$\frac{\partial f}{\partial x_{ij}^{l}}=\frac{\partial f}{\partial y_{ij}^{l}}\frac{\partial y_{ij}^{l}}{\partial x_{ij}^{l}}=\frac{\partial f}{\partial y_{ij}^{l}}\frac{\partial \left(\sigma (x_{ij}^{l})\right)}{\partial x_{ij}^{l}}=\frac{\partial f}{\partial y_{ij}^{l}}\sigma^{\prime }\left(x_{ij}^{l}\right)$$

where σ(x) is the activation function.

The correction of the weights is back-propagated over the network, however pooling layer does not take part in the training. Therefore, after calculations for fully-connected layers we come to the convolutional layer. The information gradient value for the convolutional layer is calculated using the propagated signal as:

(32) $$\begin{array}{cc} \frac{\partial f}{\partial y_{ij}^{l-1}} & =\sum_{a=0}^{m-1}\sum_{b=0}^{m-1}\frac{\partial f}{\partial x_{\left(i-a)(j-b\right)}^{l}}\frac{\partial x_{\left(i-a)(j-b\right)}^{l}}{\partial y_{ij}^{l-1}}=\\ & \sum_{a=0}^{m-1}\sum_{b=0}^{m-1}\frac{\partial f}{\partial x_{\left(i-a)(j-b\right)}^{l}}\omega_{ab} \end{array}$$

The formula from Equation (32) takes the final form:

(33) $$\frac{\partial x_{\left(i-a)(j-b\right)}^{l}}{\partial y_{ij}^{l-1}}=\omega_{ab}$$

## 4. Hybrid Architecture for Object Detection

**Figure 2:**
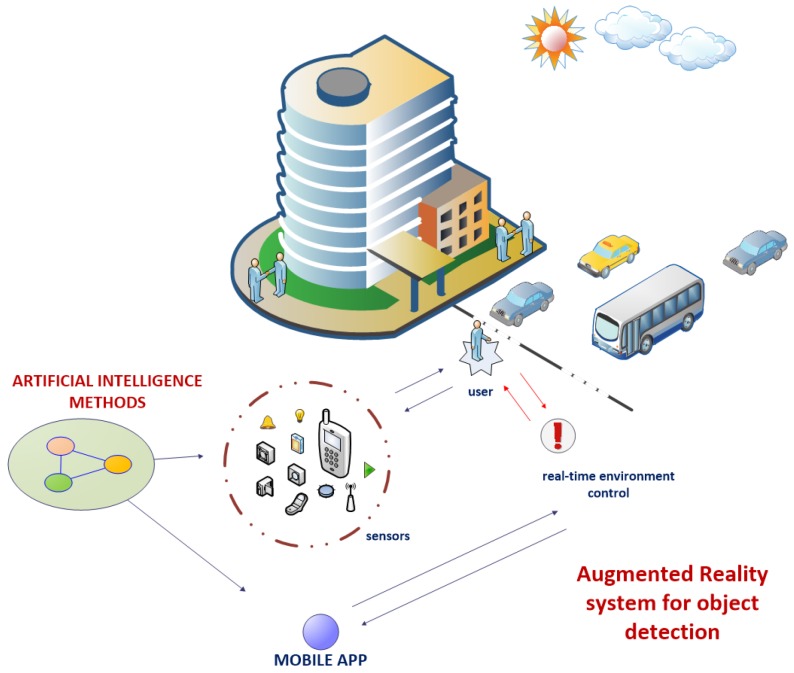
Sample presentation of the idea for an augmented reality detection system which by the use of proposed deep learning techniques evaluates readings from sensors in real time.

**Figure 3:**
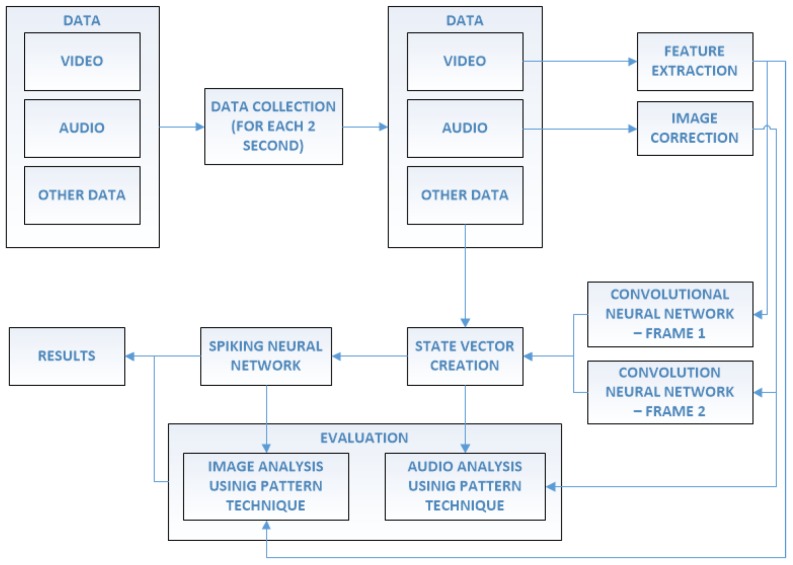
Visualization of the proposed architecture for data analysis in order to inform system users about possible obstacles.

The data obtained by the sensors in the device is complex. We have not only numerical values but also graphical samples. Very large amounts of different types of information become a big problem for one classifier, so we suggest using a hybrid architecture based on a variety of machine learning techniques. The hybrid composition we have used in the project is oriented around efficiency to get the best results. The proposed architecture operates on the flow of information between different classifiers and mechanisms, like SNN and CNN used for evaluation of sounds and images. In addition to these neural techniques we have also used other to compose a complex evaluation system for the proposed augmented reality technology. The sample visualization of the operating scheme is presented in Figure 2 and Figure 3.

### Hybrid Architecture

Incoming information cannot be interpreted separately, but all of indications need to be processed as complex data for the extraction of important features. Incoming data are composed of video, audio, and some numerical values from other sensors. The main problem is with the first two types of data—video and audio. Both types of data need to be reduced to decrease the number of calculations. For that purpose, we take only one frame every two seconds. The same is done with audio, where the spectrogram is made for these particular moments using Equation (12). Other data are selected ad hoc to obtain the specific data according to extracted frame.

On obtaining specific data, they are processed in different ways to get the most accurate evaluation results. Video is processed based on the method described in Section 2.1 and as a result we get a fragment of the image with a potential obstacle which is analyzed with Equation (9). These images are transferred to the CNN system. Sound is processed almost in the same way, in another CNN system with samples described exactly in the same way. As a result, from both networks the numerical values are returned—these networks are trained with data which contain images described with the value of 1 as an obstacle or 0 otherwise.

Both networks returned two numerical values as the responses about potential obstacles in the presented samples. Gathering these with all values received from other sensors, it is possible to create a state vector that describes situation around the user using the input data in the following manner:

(34) $$\left[x_{dev},y_{dev},z_{dev},v_{temp},v_{humi},v_{alt},i\right]$$

where $x_{dev}$, $y_{dev}$ and $z_{d}ev$ are important values for the obstacle detection captured from the recorded point of the camera. However, in the system we assume that if the camera is working and records the sky, the user should not be notified of the detected obstacles in the form of clouds. The values $v_{temp}$, $v_{humi}$, $v_{alt}$, $v_{moi}$ are the current numerical values from other sensors. More specifically, we record them from thermometers, hygrometers, and altimeters. An obstacle is marked as i∈{0,1} where 0 means no obstacle and 1 means that there is an obstacle in the area recorded by the devices.

To increase the precision, obtained results are used in next step of the proposed architecture. The spiking neural network returns the decision about a potential obstacle evaluating received information from other networks and sensors. This work is perfect for SNN, since using the model from Section 3.1 we can evaluate user state in real time. Unfortunately, this decision might be incomplete and sometimes wrong. To make sure that it is a correct result, another classifier verifies this decision based on the image from camera. The final verification is made by analyzing frames with the respect to output decision.

Suppose that the returned decision was 1 which means there was an obstacle. It should be easy to find it in the given frames. We propose the use of template technique, which means matching of the given data to the pattern—in this case, it is the obstacle. The key points in each of input frames should create a shape that can be analyzed in both frames. In the second one, the shape should be not only bigger but also more precisely visible. As “precise” we have in mind the acutance value for a random square region placed between the key points. Assume that the region size is q×q and it is converted to grayscale using Equation (13). Each pixel in the grid has 8 or 24 neighbors (which can be marked as ΔI, and the number of neighbors depends on the size of neighborhood). The difference between the center and one of the neighbors can be calculated as ΔX. By ΔI we understand the difference between the center and the neighbors in the gray scale value of the pixels, where $I_{0}$ is the double value of the squared mean value of the image. All these values are used to define acutance Ξ as:

(35) $$\Xi =\frac{1}{I_{0}}\sum \left[{\left(\frac{\Delta I}{\Delta X}\right)}^{2}\cdot \frac{1}{n}\right]$$

Hence, the defined acutance value can be used in the template matching evaluation processes based on the input video frame. The obtained important cluster should be repeated on the next frame with higher acutance. In case, when the cluster is in both frames, but the acutance value is lower, it means that the obstacle moves away. Of course, the same thing is done with voice, but here we search only for a repeating beep (a sound issued by privileged vehicles) which can be seen on video frames because of the long distance.

## 5. Data Collection

We captured data from three different users with various devices. The motivation for this was to obtain data of different quality to allow analysis of our system on the data from various perspectives. Each user had to record 40 different movies, with a minimum duration of one minute in an attempt to approach any obstacles in different weather conditions. During the recording processes a simple program was required to record the data from various sensors to the file with a time stamp. Recorded time was important for further matching of the information to the captured video files. Stored data from the first user gave 2160 frames (taking 1 frame for each two seconds of the video), 1560 frames from the second user, and 2904 frames from the third one.

## 6. Experiments

The extensive detection architecture has many different parameters that need to be controlled to obtain the highest accuracy. Each network was trained to different error values, which are 0.1, 0.01, and 0.001. Moreover, the training set was composed of 2000 frames with information from other sensors. The rest of the collected data was selected and used for the verification of correctness of the proposed architecture. In Table 1, Table 2 and Table 3 the average correctness is presented in the terms of neural network error value.

For the best correctness, we evaluated this architecture by the use of classic measures like TP (true positive), TN (true negative), FP (false positive), and FN (false negative). These values allow the calculation of more sophisticated parameters for the system: accuracy Γ, Dice’s coefficient Λ, overlap Ψ, sensitivity Υ, and specificity Φ defined as:

(36) $$\Gamma =\frac{TP+TN}{TP+TN+FP+FN}$$

(37) $$\Lambda =\frac{2TP}{2TP+FP+FN}$$

(38) $$\Psi =\frac{TP}{TP+FP+FN}$$

(39) $$\Upsilon =\frac{TP}{TP+FN}$$

(40) $$\Phi =\frac{TN}{TN+FP}$$

**Figure 4:**
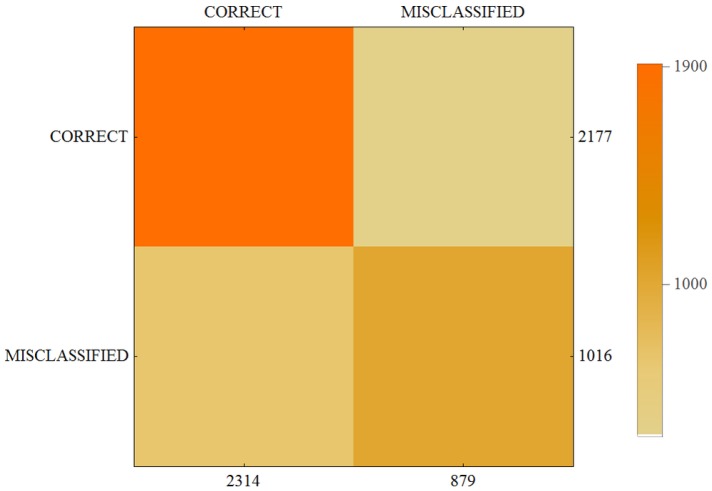
Confusion matrix for User 1.

**Figure 5:**
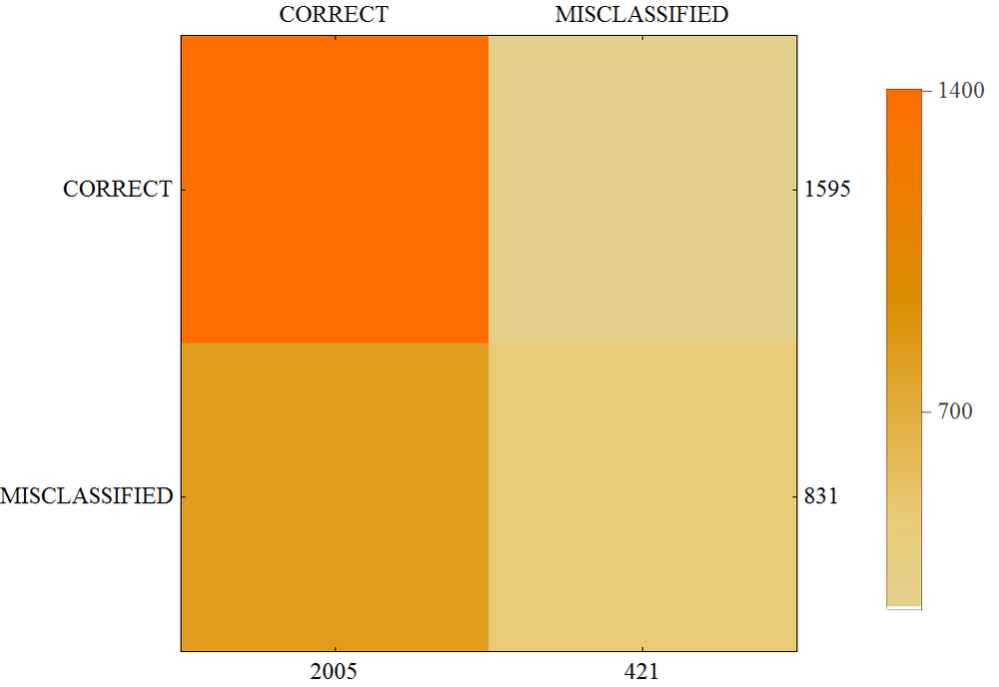
Confusion matrix for User 2.

**Figure 6:**
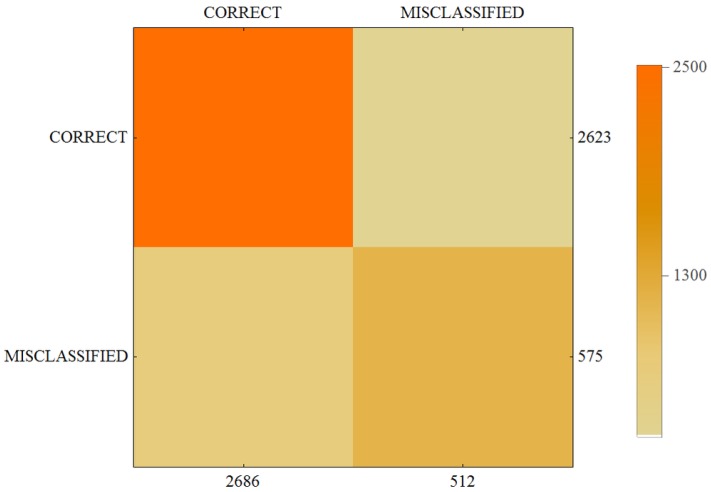
Confusion matrix for User 3.

The results of our calculations are presented in Table 4 and in the form of a confusion matrix on Figure 4, Figure 5 and Figure 6.

### Conclusions

**Figure 7:**
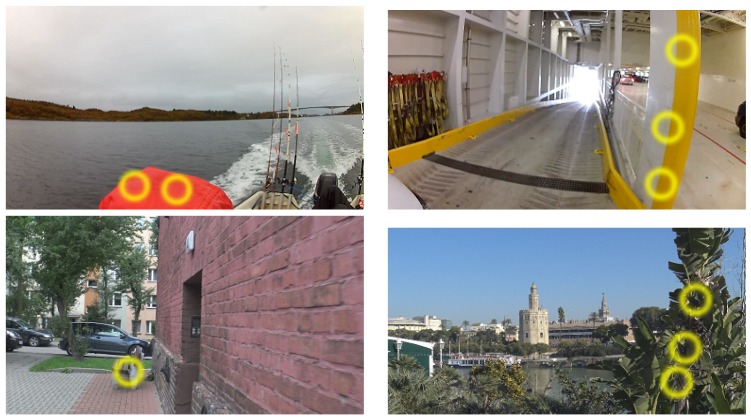
Sample frames from selected video files recorded with different devices in different weather conditions. Yellow circles indicate detected obstacles. The users were recording in various conditions: from moving vehicles with low visibility (User 1), in corridors and buildings with various levels of lighting (User 2), and in an open city space with good visibility and lightness (User 3).

For each of the three users the average correctness of evaluation is high. The best results were achieved by User 3. These recordings were done in a city during sunny day with no clouds and high visibility. The measurements were performed by walking in the streets, crossing, passing by, etc. Results show that for this kind of detection the system was able to achieve about 70% correctness. The second type of detection was measured by User 2. The recordings were done in corridors, buildings, inside constructions, etc. Conditions were independent of the weather outside, and some of the rooms and corridors were well lighted, but in others the visibility was not so good. The results of detection for User 3 reflect these conditions. The average correctness is lower but still shows that the proposed system works well. Recordings by User 1 were done from moving vehicles like cars and small boats. Weather conditions were an additional disadvantage since as we can see from Figure 7 we had restricted visibility and lightness. The objects were moving with a motor speed that implied additional difficulties for the system. Average correctness is about 50%, however the results show potential for improvement. Confusion matrices confirm that proposed solution has a high potential for further development. We can say that proposed complex system works well and it was possible to train the system with high efficiency even for three users. Therefore, for many more users working in various environments and by using additional sensors we can achieve a boost in the proposed technology.

While the results show great accuracy and many possibilities for practical applications, the number of different objects that can be encountered in reality is, unfortunately, still a problem. There is a situation where the technique will not be able to extract the features of objects, which will make the solution unusable. An example is poor recording quality, which can occur especially at night, in fog, or during heavy rain. We have also noticed that the speed of rotation of the camera must be stable. The system is not able to recognize objects if the rotation is fast. These are the main drawbacks that we have encountered in this stage of the project.

On the other hand, the solution has many positive aspects. We can use it as a system that helps older people to walk in the city, cross streets, etc. The solution is very simple in its concept, so by having a simple set of electronic devices that can provide measures of the environment in real time we can evaluate the situation and actively support older people.

## 7. Final Remarks

Increasingly practical use of augmented reality is leading to developments in technology with many possible applications. This article presents an opportunity to analyze the environment through various sensors to avoid collisions with approaching objects. This kind of technology has many uses, e.g., to help older people to cross a street, assist safer driving, or simply in supervision. For this purpose, a special architecture has been proposed which uses, among other things, deep learning algorithms and a dedicated method of data extraction from the samples obtained in real time. The proposed solution was tested and evaluated in terms of advantages and disadvantages of possible implementation in practice.

In future research, we will focus on the possibility of implementing this solution with low exposure so that the device would not be overloaded with too many operations.

## Figures and Tables

**Table 1:** Average correctness for various neural compositions applied for User 1. CNN: convolutional neural network; SNN: spiking neural network.

Error Value			Average Correctness
CNN Frames	CNN for Audio	SNN	
0.1	0.1	0.1	32%
0.1	0.1	0.01	35%
0.1	0.1	0.01	36.5%
0.1	0.01	0.1	38%
0.1	0.01	0.01	39%
0.1	0.01	0.001	39.5%
0.1	0.001	0.1	41%
0.1	0.001	0.01	39%
0.1	0.001	0.001	44%

**Table 2:** Average correctness for various neural compositions applied for User 2.

Error Value			Average Correctness
CNN Frames	CNN for Audio	SNN	
0.01	0.1	0.1	41%
0.01	0.1	0.01	43.5%
0.01	0.1	0.01	44%
0.01	0.01	0.1	43%
0.01	0.01	0.01	46%
0.01	0.01	0.001	49%
0.01	0.001	0.1	48.5%
0.01	0.001	0.01	53%
0.01	0.001	0.001	55%

**Table 3:** Average correctness for various neural compositions applied for User 3.

Error Value			Average Correctness
CNN Frames	CNN for Audio	SNN	
0.001	0.1	0.1	63%
0.001	0.1	0.01	64.5%
0.001	0.1	0.01	66%
0.001	0.01	0.1	62%
0.001	0.01	0.01	68%
0.001	0.01	0.001	71%
0.001	0.001	0.1	65%
0.001	0.001	0.01	76%
0.001	0.001	0.001	79%

**Table 4:** The results of user verification for the proposed methodology.

User	*TP*	*TN*	*FP*	*FN*	Γ	Λ	Ψ	Υ	Ψ
1	1912	614	265	402	0.79	0.85	0.74	0.82	0.7
2	1405	231	190	600	0.67	0.78	0.64	0.7	0.54
3	2512	401	111	174	0.91	0.95	0.9	0.94	0.78
Average	1943	415.33	188.67	392	0.79	0.86	0.76	0.82	0.68
